# Quantification of protein-protein interactions in highly denatured whey and potato protein gels

**DOI:** 10.1016/j.mex.2021.101243

**Published:** 2021-01-26

**Authors:** Caren Tanger, David J. Andlinger, Annette Brümmer-Rolf, Julia Engel, Ulrich Kulozik

**Affiliations:** Chair for Food and Bioprocess Engineering, Technical University of Munich, Weihenstephaner Berg 1, Freising, Bavaria 85354, Germany

**Keywords:** Solubility, Hydrophobic interactions, Disulfide bonds, Electrostatic interactions, Sodium dodecyl sulfate, Dithiothreitol

## Abstract

Understanding the stabilizing protein interactions in protein gels is of high importance for food- and biotechnology. Protein interactions in protein gels can help to predict hardness, deformability and other gel parameters. Currently there are two types methods used. One is to use protein interaction blocking agents and the other is to dissolve the gel in different buffer systems, which cleave the interactions. The first method alters the gelling mechanism, which is why the second method is the preferred one. However, currently published methods are often only suitable for specific gel systems as for example weakly bound protein gels. In this paper, a method is introduced, which is suitable for highly denatured whey and plant protein.

• Suitable for strongly cross-linked whey protein and plant protein gels
• Stronger buffer system to ensure cleavage of all protein interactions
• More reproducible and simplified crushing of the gel without the introduction of uncontrolled shear stress excessively affecting the analysis of chemical bonds

## Graphical abstract

**Figure:**
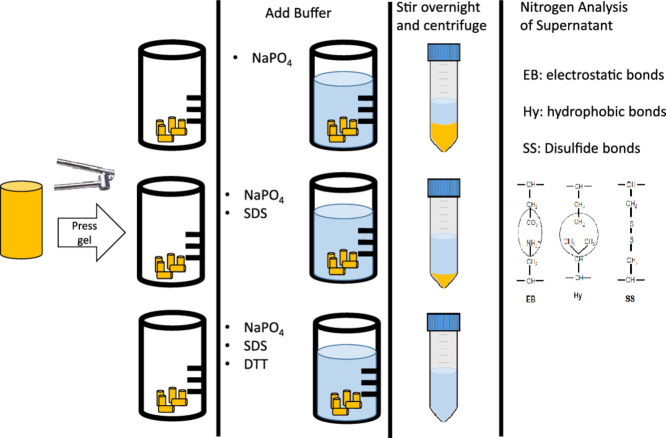

Specifications Table

**Table:** 

Subject Area	Chemistry
More specific subject area	*Protein analysis*
Method name	*Protein interaction assay*
Name and reference of original method	Keim and Hinrichs [1]Influence of stabilizing bonds on the texture properties of high-pressure-induced whey protein gels. In: International Dairy Journal 14, S. 355-363
Resource availability	• Sodium phosphate • Sodium dodecyl sulfate • Dithiothreitol • Shaker or magnetic stirring plate • Garlic press • Centrifuge • Nitrogen analysis according to Dumas

## Method details

### Background information and applicability of the method

Elevated temperature, pressure, organic solvents and other conditions are known to denature proteins. Usually, this is accompanied by unfolding of the protein. During unfolding the hydrophobic core and buried reactive amino acid groups as well as buried thiol groups get exposed and proteins can react with each other. This leads to the formation of aggregates and gels. The interactions can be non-covalent (hydrophobic, electrostatic interactions) or covalent (disulfide bonds). The type of interaction determines the textural properties of a protein gel. By controlling the protein interaction by pH and other process parameters the structural properties gels from dairy and plant proteins can be manipulated [2,3]. This is of great importance in the food industry, where proteins are used as structuring agents, next to increasing the nutritional value. One can change the process parameters and analyze the outcome by texture and rheological measurements. However, this approach does not provide in depth information about the types of bonds stabilizing the gel. This additional information is of increasing interest with the advance of plant proteins in the food industry. Animal protein are progressively substituted by plant proteins as structuring agents. However, animal and plant proteins differ greatly in their molecular structure. Examples are amount of free thiol groups, intramolecular disulfide bonds, molecular weight and surface hydrophobicity [4,5]. Therefore, they also react differently on triggers inducing gelation and the resulting gels show large differences in textural properties [6]. With this in mind, it is necessary to have a method available for analyzing the stabilizing bond interactions in animal protein gels and plant protein gels to tailor gel properties. This will allow more insights in the gelation behavior and gelation mechanisms of animal and plant protein and provides the option to modify processing parameters to obtain desired gel structures in a targeted way.

In literature, there are two main methods to determine the stabilizing protein interactions in a gel: Blocking of protein interactions during gelation and analyze resulting textural properties (1) and measuring the protein/nitrogen solubility of the produced gel in different buffers cleaving specific stabilizing protein interactions (2).

The use of blocking substances can seriously alter the gelation mechanism. For example N-Ethylmaleimide (NEM) is used to inhibit the reaction of thiol groups [7,8].

However, NEM was also shown to promote hydrophobic interactions in soy bean globulins [9] and β-lactoglobulin [10]. To determine stabilizing protein interactions in a gel, it is therefore preferred to determine the protein/nitrogen solubility in different buffer systems cleaving specific stabilizing protein interactions after gel formation has occurred. The salient buffer systems applied so far include cleaving agents such as sodium dodecyl sulfate (SDS) [1,^6^,11], urea [6,[11], [12], [13], β-mercaptoethanol [6,^11^,13] and dithiothreitol (DTT) [1,11]. These substances are known to disrupt hydrophobic (SDS and urea) and disulfide bonds (DTT and β-mercaptoethanol). This approach is reported in literature as suitable for different protein system such as egg protein gels [6], sardine muscle gels [13], pea protein gels [12], whey protein gels [6,11], lupine protein gels, soy protein gels and leaf protein (RuBisCO) gels [6]. Different cleaving agents were used by the different authors. However, differences in the properties of the cleaving agents have to be considered. β-mercaptoethanol is toxic and its disulfide reduction potential is lower compared to DTT [14]. Urea contains high amount of nitrogen, which can interfere in nitrogen content determination. Because of this, SDS and DTT are preferred as cleaving agents. The evaluation of the solubility can be either binary [6] (did the gel dissolve or not) or the amount of solubilized nitrogen can be used to provide semi-quantitative information on the contribution of each type of protein interaction [1,[11], [12], [13]. The semi-quantitative approach is preferred, because it offers the possibility of setting the individual protein interactions in relation. For example, it could be shown that an increase in protein stabilized through disulfide bonds correlated with an increase in gel strength and other rheological parameters of pressure induced whey protein gels [1]. This was possible as the changes from protein stabilized through disulfide bonds from 20% to 90% could be measured. With the binary approach, such fine differences could not have been detected.

Several methods are available for quantification of solubilized nitrogen/protein content as a base for determining the soluble protein content in the serum after cleavage of certain types of bonds. Three of these methods were recently used for quantification of stabilizing bonds, the Lowry method [12,13], absorbance at 280 nm (spectrophotometric) [11] and the Dumas method [1]. It has to be considered that the cleaving agents chosen have an effect on the nitrogen/protein content determination and vice versa. The Lowry protein assay is a biochemical assay using colorimetric techniques. The biggest disadvantages of the Lowry method are the interferences of buffer and protein with the reactive agent. This can lead to inaccuracies (physico-chemical effects, sorption) [11]. Physical interference refers to macroscopic particles. They interfere in the light scattering during photometrical absorption measurement. Physical interference also plays a role in spectrophotometric measurements. In case of DTT as the cleaving agent, the oxidized form of DTT has the same absorption maximum as the one of protein [15]. This makes the method unnecessarily prone to experimental error. For two of these methods, Lowry and spectrophotometric measurement, a calibration curve is needed and this is highly labor intensive. The third method mentioned was the Dumas method. There, the nitrogen content in the buffer system is determined by controlled combustion of a sample. Therefore, there is no interference of a chemical reagent with the protein or buffer. An additional advantage is the reproducibility and the high throughput. This is the reason why the Dumas method is the method of choice of many laboratories to determine nitrogen/protein content. A disadvantage of the Dumas method, however, is that the total nitrogen content of the sample is determined. This means that nitrogen containing substances in the buffer are also measured as protein. This would be the case for urea and the common buffer substance 2-Amino-2-(hydroxymethyl)propane-1,3-diol (TRIS) leading to a high background noise. However, the method of Dumas can be considered advantageous. However, buffers with nitrogen containing substances should be avoided. The Dumas method was utilized by Keim and Hinrichs for the determination of pressure induced whey protein gels [1] and acid, rennet and pressure induced milk protein gels [16] with some limitations or even restrictions regarding highly thermally denatured protein.

The aim of this work was to modify a protein interaction assay, which can be applied to both highly denatured animal derived proteins (especially whey proteins) and plant proteins. The determination of protein interactions should be semi-quantitative. Furthermore, a high reproducibility and high throughput were aimed at.

Concluding from this, the method of Keim and Hinrichs [1] was the most suitable for modification. It is a semi-quantitative method using the Dumas method for nitrogen quantification and the buffer system contains SDS and DTT as cleaving agents. The method was used for whey protein with a low degree of denaturation [1,17], where protein interaction was weak. In order to extend the method and to make it suitable for highly denatured whey and plant protein with strong protein interactions, adjustments to the buffer systems, dissolving method and dissolving parameters had to be made. The proposed and validated changes are discussed in detail below.

### Explanation on changes made

The following buffer system was used by Keim and Hinrichs. Buffer S1 was composed to cleave all hydrophobic and electrostatic interactions and contained a TRIS-Acetate buffer with SDS. Buffer S2 was composed to cleave all hydrophobic and electrostatic interactions and disulfide bonds and contained a TRIS-Acetate buffer, SDS and DTT. In theory, the gel should completely dissolve in buffer S2. Buffer S3 was composed to cleave all electrostatic interactions and contained a sodium phosphate buffer with NaCl. In later works the authors added two new buffers, which were able to cleave calcium bridges (D) and non-specific bonds (H) [16]. This addition made the method suitable for pressure-induced, heat-induced and rennet-induced milk protein gels. TRIS contains nitrogen, which leads to a high background noise during nitrogen measurement. The authors probably used a TRIS-Acetate buffer, because it does not interact with casein micelles. Casein micelles are present in milk protein gels. Phosphate buffers, on the other hand, are known to severely influence the casein equilibrium and alter micelle structure and composition [18]. However, caseins are not present in whey protein or plant protein. In order to reduce the background noise, the TRIS-Acetate buffer was substituted by a phosphate buffer. No differences were found between the usage of TRIS-Acetate and phosphate buffer for whey and plant protein gels. The buffers S1 and S2 were not able to cleave all hydrophobic interactions and disulfide bonds in highly denatured whey and plant proteins. Therefore, the concentration of SDS and DTT had to be increased. Additionally, the pH was increased to pH 7.5 to increase the reducing ability of DTT. Furthermore, stirring time was increased to a minimum of 16 h. These were found to be optimal condition for DTT and SDS to cleave all protein interactions. Stirring temperature was set to room temperature to avoid crystallization of SDS at low temperatures.

**Fig. 1:**
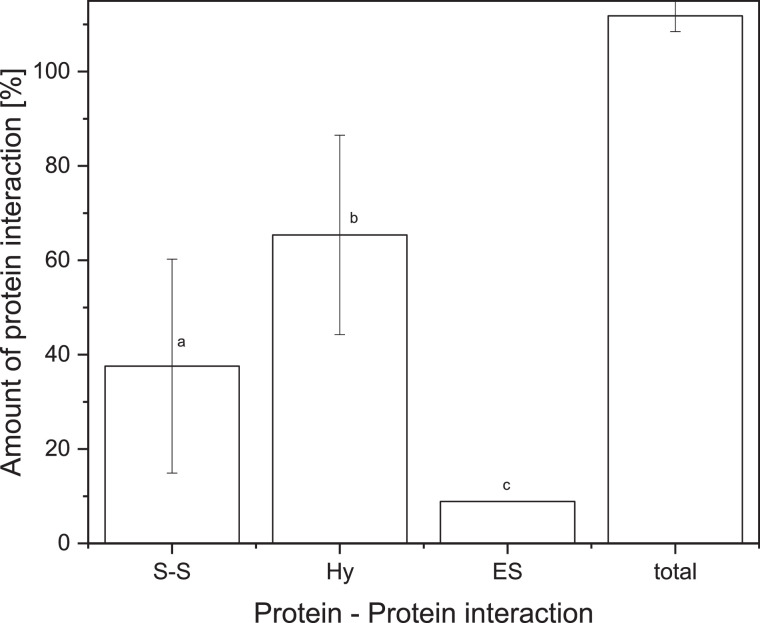
Protein interactions of a WPI gel prepared at pH 7 at 85°C crushed with an ultra-turrax, determined in six-fold. Error bars depict standard deviation. a–c different letters indicate significant differences between samples (*P* < 0.05).

The cleaving agents can only work on the exposed surface of the gel. They cannot penetrate into the inside of the gel. In order to increase the exposed surface of the gel Keim and Hinrichs [1] as well as Gómez-Guillén et al. [13], Felix et al. [12] and Shimada and Cheftel [11] used an ultra-turrax to crush the gel in the buffer. The ultra-turrax can shear the gel in small particles, which increases the exposed surface. However, it also induced an uncontrollable shear stress destroying bonds prior to the analysis in an uncontrolled or excessive way. This can lead to misconceptions regarding the types of bonds stabilizing a gel. Whey protein gels produced at pH 7 and 15 % protein concentration heated for 30 min above denaturation temperature led to very hard gels. The gel either got partially stuck in the case of the ultra-turrax or was not comminuted at all, thus not allowing the buffer systems to enter the gel sample. This leads to a low reproducibility. The result of a six-fold measurement of such a gel using an ultra-turrax is shown in Fig. 1.

The error bars depict the standard deviations, which are very high (>15 %). Although, the amount of disulfide bonds and hydrophobic interactions show a significant difference, the *p-*value was high with *p=*0.0239. There was also a significant difference between disulfide bonds and electrostatic interaction. Again the *p-*value was high with *p=*0.0210. Therefore, the method was not sufficiently reproducible. Because of this, it was chosen to replace the ultra-turrax as a device for mechanical sample pre-treatment. The requirements were that the crushing method was reproducible and it should not destruct the gel beyond the level required to allow the cleaving buffering systems to diffuse into the gel in an acceptable short period of time. The requirements were fulfilled by using a garlic press. Pressing the gel through the garlic press resulted in laces of 2 mm diameter. The laces were then chopped in smaller cylinders. The cleaving agents were thus able to reproducibly cleave all protein interactions.

### Description of modified protein interaction assay

For the method development, heat set protein gels at different pH values from patatin rich potato protein isolate and whey protein isolate were created. Commercial patatin rich potato protein isolate (PPI) powder (Solanic 200), was kindly provided by AVEBE (Veendam, The Netherlands). The protein powder had a protein content of 88.6% (w/w). Commercial WPI (BiPRO^TM^) powder from Agropur Dairy Cooperative (Saint-Hubert, Longueuil, Canada) was obtained. The protein powder had a protein content of 90.9 % (w/w). The protein content was determined using the method of Dumas with an accuracy of ± 0.1% (w/w) (Vario MAX CUBE, Elementar Analysensysteme GmbH, Hanau, Germany). The Dumas factor was 6.38 and 6.25 for WPI and PPI, respectively. However, the Dumas factors are not necessary to determine soluble nitrogen.

#### Preparation of buffer systems

Three buffers were prepared. The detailed composition of the three buffers can be found in Table 1. Buffer B1 dissolves proteins bound by electrostatic and non-specific interactions. Buffer B2 additionally dissolves Proteins bound by hydrophobic interaction due to the addition of SDS. Buffer B3 additionally dissolves proteins bound by covalent disulfide bonds due to the addition of DTT.

**Table 1:** Composition of the three different buffers used in this study.

Buffer system	NaH_2_PO_4_ /Na_2_HPO_4_ [mol/L]	SDS [g/L]	DTT [g/L]	pH
B1	0.05	-	-	7.5
B2	0.05	2	-	7.5
B3	0.05	2	15	7.5

Note: Higher concentration of DTT and SDS, compared to Keim & Hinrichs [1], were chosen to fully dissolve strongly bound globular proteins. Even higher concentrations of SDS were also tested (up to 10 g/L). However, this resulted in the formation of filaments of some covalent linked protein gels, which could not be separated through centrifugation.

### Dissolution of gel in buffer system

1. Press gel through a garlic press, for homogenization and size reduction
2. Weigh 0.5 g of crushed gel in each of three 50 ml glass beakers
3. Label the glass beakers B1, B2, B3
4. Pour 20 g buffer of the respective buffer in each in beaker

Note: 0.5 g gel does refer to a gel with 10 –15 % protein content. Thus, the protein to buffer ratio is 0.01–0.015: 4. If the nitrogen content gets too high, the buffer is not able to cleave all protein – protein interactions in the gel.

### Mixing

Stir overnight at room temperature with a magnetic stirrer

Note: Dissolving in centrifuge tubes and using a shaker instead of a beaker and a magnetic stirrer does also work and allows dissolving and centrifuging in the same tube increasing the reproducibility of the process.

### Determination of soluble nitrogen content

1. Centrifuge stirred samples at 10 000 g at 20°C for 20 min
2. Separate supernatant from pellet. The pellet can be discarded.
3. Determine % nitrogen content in supernatant by the Dumas method
4. Determine also the % nitrogen content of the original gel by the Dumas method

Note: An SDS-PAGE of the supernatants of the gel dissolved in the three buffers can give an indication of which proteins are engaged in the different protein – protein interactions. Some pellets were very soft and the supernatant had to be removed with a pipette to avoid mixing between pellet and supernatant.

### Quantification of protein interactions

First, the concentration of protein bonds that are cleaved by the different buffer systems ($C_{n,bond,Bx})$ has to be calculated. This is shown in Eq. (1):

(1) $$C_{n,\;bond,\;Bx}=\;\frac{\left(m_{S}+m_{gel}\right)}{m_{gel}}*C_{n,\;sup,\;Bx}$$

With $m_{S\;}$being the initial mass of the buffer (20 g), $m_{gel}$ being the mass of the gel (0.5 g) and $C_{n,sup,Si}$ being dissolveld nitrogen in the supernatant in [%], determined by the method of Dumas.

Buffer B1 cleaves electrostatic protein-protein interactions and hydrogen bonds [P(ES)]:

(2) $$\frac{C_{n,\;bond,\;B1}}{C_{n,gel}}=\;P\left(ES\right)$$

With $C_{n,gel}$ being the nitrogen content in [%] of the analyzed gel. P describing the relative amount of protein bound by this protein interaction. ES is abbreviated for electrostatic bonds including hydrogen bonds

Buffer B2 cleaves all electrostatic [P(ES)] and hydrophobic protein-protein interactions [P(Hy)]. Therefore, it can be said that:

(3) $$\frac{C_{n,\;bond,\;B2}}{C_{n,\;gel}}=\;P\left(ES\right)+P\left(Hy\right)$$

with Hy being short for hydrophobic interactions.

Buffer B3 cleaves all protein-protein interactions, including disulfide bonds [P(SS)]. Therefore, it can be said that:

(4) $$\frac{C_{n,\;bond,\;B3}}{C_{n,gel}}=\;P\left(ES\right)+P\left(Hy\right)+P\left(SS\right)$$

With SS being short for disulfide bonds.

From Eqs. (1)–(4) we can deduce following equations to calculate the amount of protein bound by the different protein interactions.

The quantity of electrostatic interactions including hydrogen bonds is given by the concentration of solubilized protein nitrogen in Buffer C divided by the protein nitrogen content of the analyzed gel:

(5) $$P\left(ES\right)=\;\frac{C_{n,bond,\;B1}}{c_{n,gel}}$$

The amount of protein nitrogen bound by hydrophobic interactions can be calculated by the difference of solubilized protein nitrogen in buffer B2 and B1:

(6) $$P\left(Hy\right)=\;\frac{C_{n,bond,\;B2}}{c_{n,gel}}-\frac{C_{n,bond,B1}}{C_{n,gel}}$$

The amount of protein nitrogen bound by disulfide bonds can be calculated by the difference of concentration of solubilized protein nitrogen in buffer B3 and B2:

(7) $$P\left(SS\right)=\;\frac{C_{n,bond,\;B3}}{C_{n,gel}}-\frac{C_{n,bond,B2}}{C_{n,gel}}$$

## Method validation

### Reproducibility of method

In order to validate the method and to test the buffer system, three different types of gels were tested six-fold. For each gel type samples for six-fold measurement were taken from the same gel. This was done to test the reproducibility of the method and not of the gel formation. Results were also analyzed statistically using a one-way analysis of variance (ANOVA) and students t-test for significant difference between the amount of protein interactions (see Fig. 3). Minimum significance was set at the 5 % level (p < 0.05). The chosen test gels should be different in their dominant protein interactions. Therefore, two different whey protein gels and a potato protein gel were tested. One whey protein gel was made by heating a 15 % whey protein solution 30 min at 85°C at pH 7. The second whey protein gel was made by heating a 15 % whey protein solution at pH 5 30 min at 70°C. The potato protein gel was made by heating a 10 % potato protein solution at pH 7 for 30 min at 70°C. Differences in protein type, heating and milieu conditions will lead to different protein interactions being dominant within the gels. First the reproducibility of the nitrogen solubilization will examined. Afterwards, the results of this will be discussed with literature findings.

**Fig. 2:**
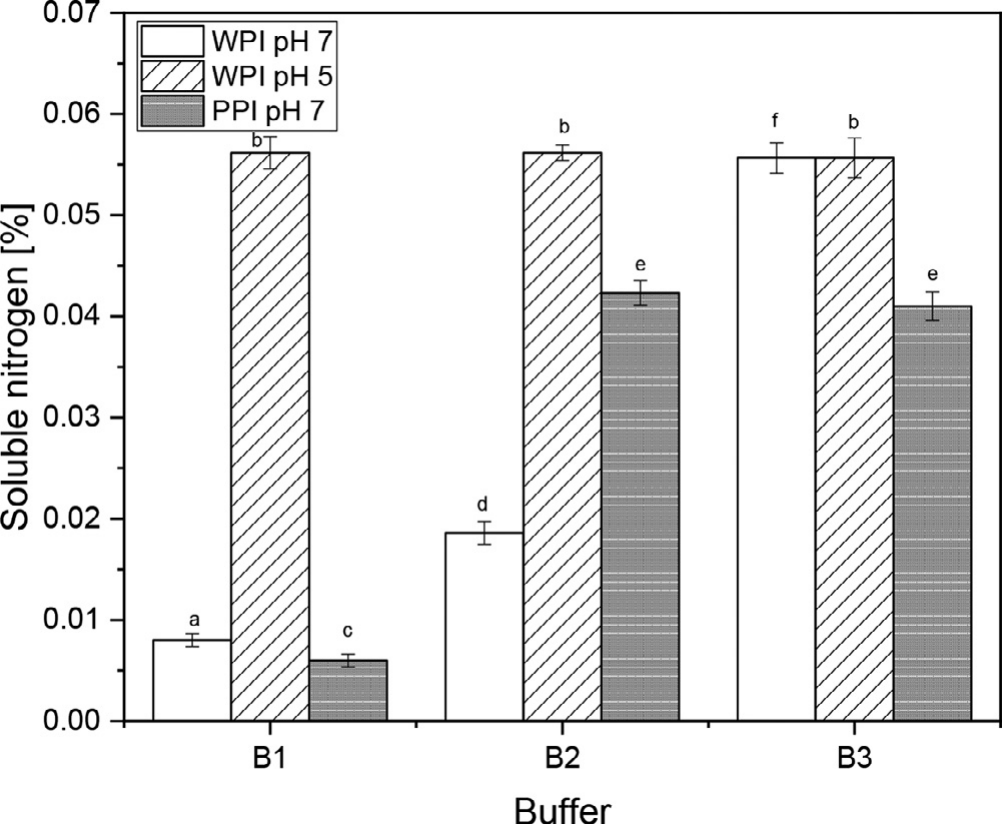
Soluble nitrogen content of the supernatant of the three gels dispersed in the three buffers (see Table 1 for composition) and centrifuged at 10,000 g for 20 min. The three gels were: a WPI gel at pH 7 heated 85°C, a WPI gel at pH 5 heated at 70°C and a PPI gel at pH 7 heated at 70°C. All gels where heated for 30 min. ^a-f^different letters indicate significant differences between samples (*P* < 0.05).

The soluble nitrogen content in the different buffers with standard deviation are shown in Fig. 2. The soluble nitrogen content already indicates, which protein interaction is dominant in the gel. For PPI only a 10 % (w/w) protein gel was used therefore less nitrogen can be solubilized. This explains the low soluble nitrogen content in buffer B3 of the PPI gel.

Most important in Fig. 2 is the standard deviation. In contrast to Fig. 1 the standard deviation is acceptably small. This implies that the changes made to the initial method of Keim and Hinrichs [1] led to a reproducible method for highly denatured whey and plant proteins.

**Fig. 3:**
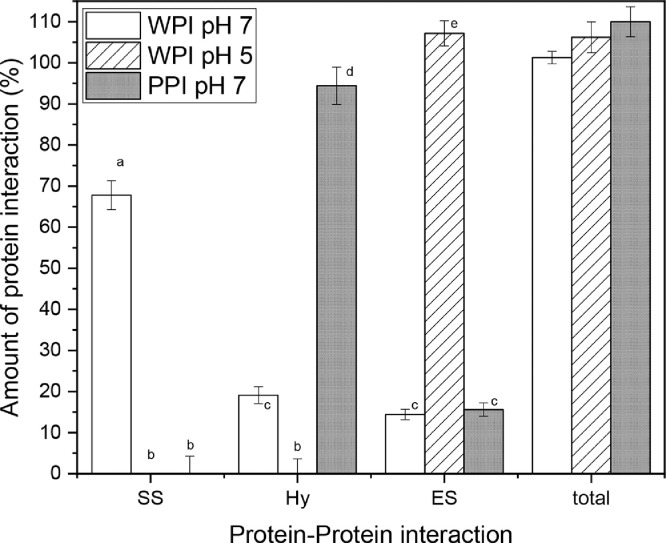
Protein interactions of a WPI gel at pH 7 heated at 85°C, a WPI gel at pH 5 heated at 70°C and a PPI gel at pH 7 heated at 70°C. All gels where heated for 30 min. ^a-e^ different letters indicate significant differences between samples (*P* < 0.05)

In the following the ability of the buffers to cleave all protein interactions is determined. First, the gels were dissolved in buffer and nitrogen content after solubilization was determined. Afterwards, Eqs. (1)–(7) were applied to the measured nitrogen solubility in each buffer. From this the relative amount of each protein interaction within the gels were obtained. The results of the calculations are shown in Fig. 3. It should be noted that the highest amount of nitrogen is dissolved in buffer B3 as this buffer cleaves all protein interactions. The total amount of protein interactions was found to be around 100 % for each gel measured independent of dominant protein interaction found in the gel. Therefore, it can be said, that the buffer system and dissolving parameters were sufficient to cleave all protein interactions. Below, the results obtained with this method were compared to literature findings using other methods.

As expected, the main protein interaction in whey protein gels, heated well above their temperature of denaturation and at pH 7, was by disulfide bonds (66 %) followed by hydrophobic interactions (19 %). Electrostatic interactions played a minor role. The formation of disulfide linked whey protein gels and the effect of pH and temperature on the gelation are described in detail elsewhere [19,20]. Monahan et al. [19] and Sava et al. [20] also found that disulfide bonds were the dominant protein interaction in heated whey protein and β-lactoglobulin by measuring the sulfhydryl group content using Ellmann reagent.

In contrast, protein interactions in the whey protein gel heated at pH 5 below denaturation temperature were mainly found to be of electrostatic nature. For these type of gels, the low electrostatic repulsion of protein monomers led to electrostatic interactions without the formation of disulfide bonds. This is in line with the finding of Sava et al. [19].

The potato protein gel was formed mainly by hydrophobic interactions. Patatin, the main potato protein, contains one free thiol group. Therefore, only two patatin monomers can be bound together by a disulfide bridge. In this case, no larger networks can be formed by disulfide bonds as compared to whey proteins which create multilateral networks by a thiol-disulfide exchange mechanism [5,21]. The lack of internal disulfide bonds in patatin leads to an extensive unfolding upon heating [22]. Upon unfolding, the hydrophobic core gets exposed and hydrophobic residues can react with each other. Several proteins can react with each other via hydrophobic interactions building a gel network. Gelation of potato protein is not dependent on the formation of disulfide bonds. This explains the dominant hydrophobic interactions in the potato protein gel.

Summarizing, the method was found to be reproducible and all protein interactions were cleaved by SDS and DTT. The dominant protein interactions of the three gels were in line with what was expected from the literature.

## Limitation of the method

**Fig. 4:**
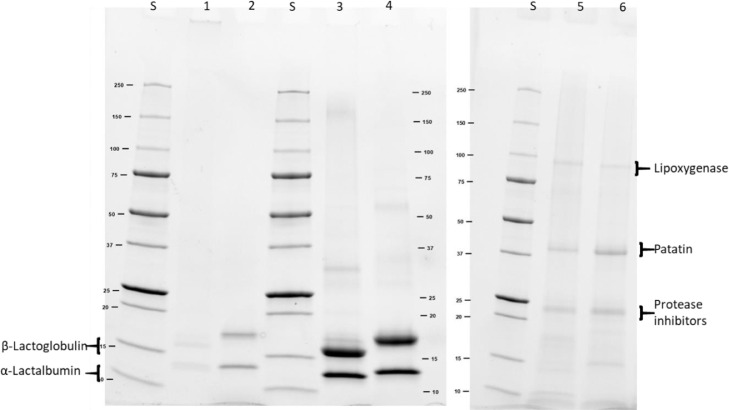
SDS-PAGE of supernatant of buffer B2 of the two WPI gels and the PPI gel. S marks the standard lane. Lane 1 and 2 are WPI gels at pH 7, non-reducing and reducing. Lane 3 and 4 are WPI pH 5, non-reducing and reducing. Lane 5 and 6 are PPI gels at pH 7, non-reducing and reducing.

The method is based on the centrifugal separation. Cleaved particles became soluble and uncleaved gel particles remained insoluble. Disulfide linked dimers or small oligomers cannot be cleaved by buffer B2. However, these protein particles are small enough to stay soluble in buffer B2. They contribute to the calculated hydrophobic interactions even though they are cross-linked by disulfide bonds. To investigate those small oligomers, an SDS-PAGE with the supernatant of buffer B2 was performed. The SDS-PAGE of the three gels in buffer B2 can be seen in Fig. 4.

Uncleaved oligomers can be found in the pocket of the SDS-PAGE. This is the case for whey protein gel prepared at pH 7 at 85°C for 30 min (Lane 1). Thus, there are aggregates bound via disulfide bonds, which are larger than 250 kDa, but small enough to stay soluble during centrifugation. The two other bands in lane 1 are monomeric β-Lg and α-La. No dimers were found. With this it can be said that there are monomeric proteins, which are bound together by hydrophobic interactions. It can also be said that there are small oligomers cross-linked by disulfide bonds. These small oligomers are connected by hydrophobic interactions forming a gel network. No bands were found for proteins above 250 kDa in the whey protein gel prepared at pH 5, with heating below denaturation temperature. Thus, no disulfide bonds were formed. In the potato protein gel, no bands above 250 kDa could be found. The band at 95 kDa could be attributed to lipoxygenase and does not disappear in a reducing SDS-PAGE. Therefore, it can be said that this gel solely relies on hydrophobic interactions. The addition of performing an SDS-PAGE with the supernatant of the three buffers provides even more information to the protein interaction assay. It gives the opportunity to separate between the protein interaction between big particles and between the proteins itself. The SDS-PAGE gel also shows a limitation of the method presented in this paper. Separation of cleaved and uncleaved proteins and particles by the buffers is based on centrifugation. Therefore, smaller aggregates, such as dimers and trimer, bound together by disulfide bonds, stay soluble in buffer B2 and add to the calculated hydrophobic interactions. They can only be made visible by non-reducing SDS-PAGE.

Note: If not all protein is dissolved in buffer B3 an investigation into the pellet by SDS-PAGE could hint at the formation of isopeptide bonds, as described elsewhere [23]. However, dissolution of the pellet in the SDS-PAGE buffer indicates that the buffer B3 is not strong enough to cleave all disulfide bonds. This was the case when WPI gels heated at pH 7 and 85°C were investigated and the DTT concentration was below 15 g/L and the protein to buffer ratio was higher than described here.

## Conclusion

In this paper a method for the determination of stabilizing protein bonds in a protein gel is presented. The method was based on an existing method for weakly bound milk protein gels and adapted. Changes to the composition of the buffer system, the crushing method of gel and dissolution parameters had to be made. It could be shown that the presented method is reproducible and suitable for highly denatured whey and plant protein gels. A limitation is that stabilizing protein bonds of small aggregates such as dimers and small oligomers could not be determined. From this, it can be anticipated that the presented method is of interest for further research in strongly bound protein gels from both animal and plant proteins. Especially for plant proteins, there is still a lot to be learned about the type of protein interactions forming aggregates and gels.

## References

[1] Keim S., Hinrichs J. (2004). Influence of stabilizing bonds on the texture properties of high-pressure-induced whey protein gels. Int. Dairy J..

[2] Nicolai T., Britten M., Schmitt C. (2011). β-Lactoglobulin and WPI aggregates: Formation, structure and applications. Food Hydrocolloids..

[3] Nicolai T., Chassenieux C. (2019). Heat-induced gelation of plant globulins. Curr. Opin. Food Sci..

[4] Delahaije R.J.B.M., Wierenga P.A., Giuseppin M.L.F., Gruppen H. (2015). Comparison of heat-induced aggregation of globular proteins. J. Agric. Food Chem..

[5] Creusot N., Wierenga P.A., Laus M.C., Giuseppin M.L.F., Gruppen H. (2011). Rheological properties of patatin gels compared with β-lactoglobulin, ovalbumin, and glycinin. J. Sci. Food Agric..

[6] Martin A.H., Nieuwland M., de Jong G.A.H. (2014). Characterization of heat-set gels from RuBisCO in comparison to those from other proteins. J. Agric. Food Chem..

[7] Sun X.D., Arntfield S.D. (2012). Molecular forces involved in heat-induced pea protein gelation: Effects of various reagents on the rheological properties of salt-extracted pea protein gels. Food Hydrocoll..

[8] Mounsey J.S., O'Kennedy B.T. (2007). Conditions limiting the influence of thiol–disulphide interchange reactions on the heat-induced aggregation kinetics of β-lactoglobulin. Int. Dairy J..

[9] Hua Y., Cui S.W., Wang Q., Mine Y., Poysa V. (2005). Heat induced gelling properties of soy protein isolates prepared from different defatted soybean flours. Food Res. Int..

[10] Xiong Y.L., Dawson K.A., Wan L. (1993). Thermal aggregation of β-lactoglobulin: effect of pH, ionic environment, and thiol reagent1. J. Dairy Sci..

[11] Shimada K., Cheftel J.C. (1988). Texture characteristics, protein solubility, and sulfhydryl group/disulfide bond contents of heat-induced gels of whey protein isolate. J. Agric. Food Chem..

[12] Felix M., Perez-Puyana V., Romero A., Guerrero A. (2017). Development of thermally processed bioactive pea protein gels: Evaluation of mechanical and antioxidant properties. Food Bioprod. Process..

[13] Gómez-Guillén M.C., Borderías A.J., Montero P. (1997). Chemical interactions of nonmuscle proteins in the network of sardine (Sardina pilchardus) muscle gels. LWT - Food Sci. Technol..

[14] Lukesh J.C., Palte M.J., Raines R.T. (2012). A potent, versatile disulfide-reducing agent from aspartic acid. J. Am. Chem. Soc..

[15] Cleland W.W. (1964). Dithiothreitol, a new protective reagent for SH Groups *. Biochemistry.

[16] Keim S., Kulozik U., Hinrichs J. (2006). Texture and stabilizing bonds in pressure-induced, heat-induced and rennet-induced milk protein gels. Milchwissenschaft.

[17] Keim S. (2005). Hydrostatisch, thermisch, säure- und labinduzierte Casein- und Molkenprotein-Gele: Stabilisierende Bindungen und Textureigenschaften.

[18] Udabage P., McKinnon I.R., Augustin M.A. (2000). Mineral and casein equilibria in milk: effects of added salts and calcium-chelating agents. J. Dairy Res..

[19] Monahan F.J., German J.B., Kinsella J.E. (1995). Effect of pH and temperature on protein unfolding and thiol/disulfide interchange reactions during heat-induced gelation of whey proteins. J. Agric. Food Chem..

[20] Sava N., van der Plancken I., Claeys W., Hendrickx M. (2005). The kinetics of heat-induced structural changes of β-lactoglobulin. J. Dairy Sci..

[21] Pots A.M., ten Grotenhuis E., Gruppen H., Voragen A.G.J., de Kruif K.G. (1999). Thermal aggregation of patatin studied in situ. J. Agric. Food Chem..

[22] Pots A.M., de Jongh H.H.J., Gruppen H., Hessing M., Voragen A.G.J. (1998). The pH dependence of the structural stability of patatin. J. Agric. Food Chem..

[23] Rombouts I., Lagrain B., Brunnbauer M., Koehler P., Brijs K., Delcour J.A. (2011). Identification of isopeptide bonds in heat-treated wheat gluten peptides. J. Agric. Food Chem..

